# Efficacy of zinc carnosine in the treatment of colorectal cancer and its potential in combination with immunotherapy *in vivo*

**DOI:** 10.18632/aging.204380

**Published:** 2022-11-14

**Authors:** Weiwei Tang, Hanyuan Liu, Xiao Li, Theng Choon Ooi, Nor Fadilah Rajab, Hongyong Cao, Razinah Sharif

**Affiliations:** 1 Center for Healthy Ageing and Wellness, Faculty of Health Sciences, University Kebangsaan Malaysia, Kuala Lumpur 50300, Malaysia; 2 Hepatobiliary/Liver Transplantation Center, The First Affiliated Hospital of Nanjing Medical University, Key Laboratory of Living Donor Transplantation, Chinese Academy of Medical Sciences, Nanjing 210000, Jiangsu, China; 3 General Surgery, Nanjing First Hospital, Nanjing Medical University, Nanjing 210000, Jiangsu, China; 4 Biocompatibility Laboratory, Centre for Research and Instrumentation, University Kebangsaan Malaysia, UKM Bangi, Bangi 43600, Selangor Darul Ehsan, Malaysia

**Keywords:** ZnC, colorectal cancer, PD1, PD-L1, immune

## Abstract

Background: A complex of Zn and carnosine, called Zinc-L-carnosine (ZnC), enjoys a wide application as part of a Zn supplement therapeutic method as well as in treating peptic ulcers. However, researches fail to confirm the biological functions possessed by ZnC as well as tumor immune microenvironment in colorectal cancer (CRC).

Methods: Cell counting kit 8(CCK8), 5-ethynyl-2'-deoxyuridine (EdU), transwell and wound healing assays were conducted to study the influence of ZnC in the proliferating, invading and migrating processes of CRC cell lines (HCT116, LOVO) *in vitro*. The antitumor activity ZnC as well as its effects on tumor immune microenvironment were then assessed using CRC subcutaneous tumors in the C57BL/6 mouse model.

Results: According to CCK8, EdU, transwell and wound healing assays, ZnC inhibited CRC cell lines in terms of proliferation, invasion and migration. ZnC could inhibit miR-570 for up-regulating PD-L1 expression. *In vivo* experiments showed that gavage (100 mg/kg, once every day) of ZnC inhibited the tumor growth of CRC, and the combination of ZnC and anti-PD1 therapy significantly improved the efficacy exhibited by anti-PD1 in treating CRC. In addition, mass cytometry results showed that immunosuppressive cells including regulatory T cells (tregs), bone marrow-derived suppressor cells (MDSC), and M2 macrophages decreased whereas CD8+ T cells elevated after adding ZnC.

Conclusions: The present study reveals that ZnC slows the progression of CRC by inhibiting CRC cells in terms of proliferation, invasion and migration, meanwhile up-regulating PD-L1 expression via inhibiting miR-570. The ZnC-anti-PD1 co-treatment assists in synergically increasing anti-tumor efficacy in CRC therapy.

## INTRODUCTION

Colorectal cancer (CRC) acts as the third cause of death related to cancer all over the world [1]. Although therapeutic agents such as molecular targeting agents have made progressions recently, e.g. bevacizumab specific to vascular endothelial growth factor-A (VEGF-A), cetuximab specific to epidermal growth factor receptor (EGFR), and encorafenib specific to murine sarcoma viral oncogene homolog B1 (BRAF), patients who had metastatic CRC (mCRC) still presented poor prognosis [2]. Hence, aggressive prophylaxis strategies and new effective therapies are needed to prevent and treat this malignancy.

In recent years, immune checkpoints (ICIs) targeting programmed death 1 (PD1) and cytotoxic T lymphocyte-associated antigen 4 (CTLA-4) have transformed the treatment landscape for various cancers, including CRC [3]. Although ICIs alone can lead to long-term remission and improved survival outcomes in certain populations, a large number of patients do not respond effectively. Immunotherapy may be one of the effective treatment strategies to improve tumor response rate and prognosis. Immunotherapy drugs can be used in combination with other drugs to enhance the immunogenicity of tumors, thereby improving their efficacy [4–6].

Zinc L-carnosine (ZnC, i.e Polaprezinc) is a chelating compound composed of L-carnosine and zinc [7]. The combination or chelation of zinc and carnosine results in ZnC which is said to have better health benefits in enhancing zinc absorption than carnosine alone due to its solubility may be because it provides a delayed tissue/extended release of zinc method [8]. In the United States, ZnC is licensed as a dietary zinc supplement and possible adjunct to promote the recovery of healthy gastric mucosa in patients with peptic ulcer disease [9]. However, there is evidence that it can also restore tissue in other parts of the gastrointestinal tract. For example, studies support its role in taste disorders, gastrointestinal disorders, skin, liver and chemotherapy-induced oral mucositis [10]. However, researches have not determined the biological functions possessed by ZnC as well as the tumor immune microenvironment in CRC. Our study investigated the proliferation, invasion, migration, and immune effect exerted by ZnC in CRC progression. The co-treatment combining ZnC and PD1 monoclonal antibody (mAb) may assist in increasing the antitumor activity, thereby helping to better understand the CRC immunotherapy.

## RESULTS

### ZnC inhibited the proliferation, invasion and migration of CRC cells *in vitro*

**Figure 1:**
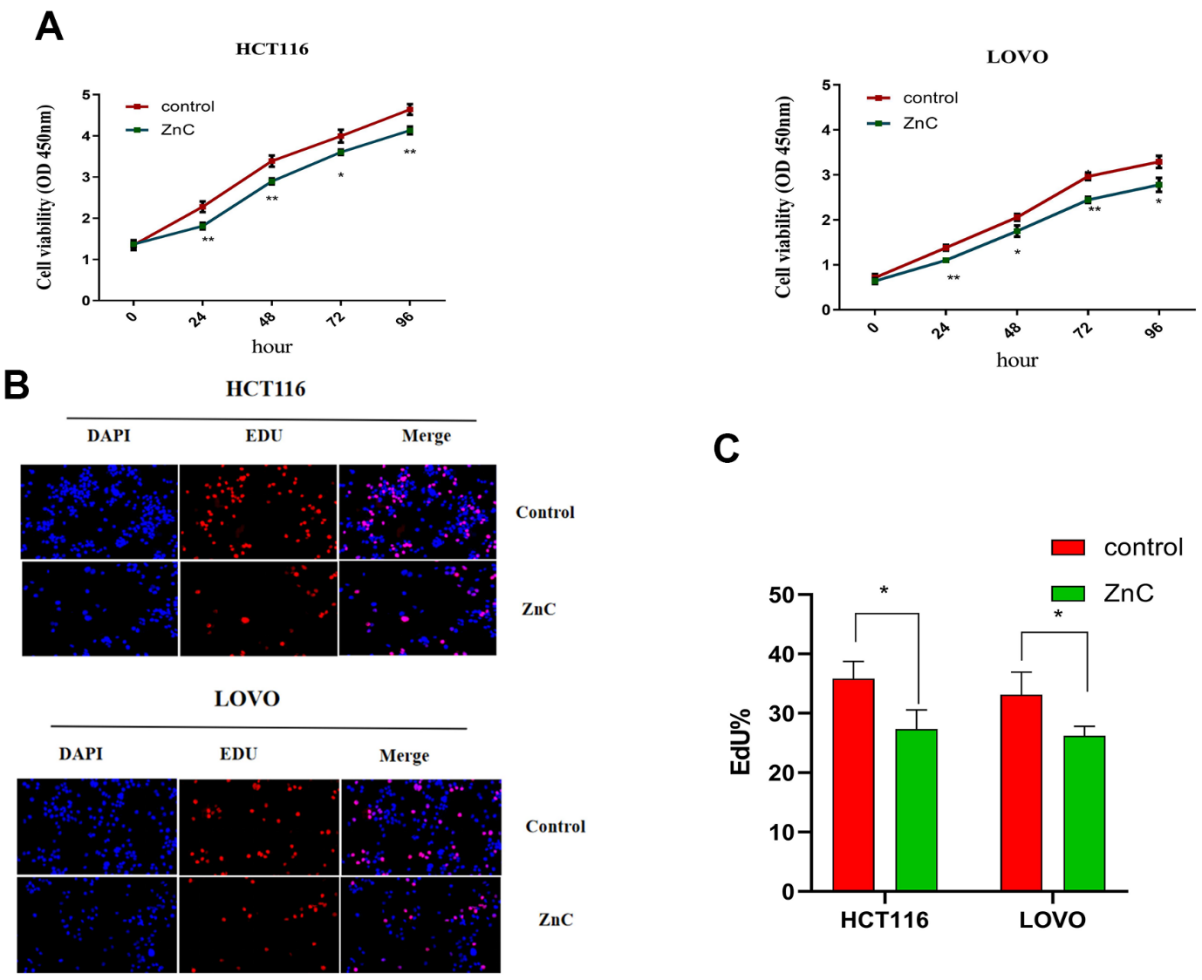
**ZnC inhibited the proliferation in CRC cell lines.** (**A**) The growth curves of CRC cells were plotted after cultured with ZnC based on CCK-8 assays. (**B**) EdU assays were performed to assess cell proliferation of HCT116 and LOVO cell lines cultured with ZnC. (**C**) EdU analysis. *p < 0.05, **p < 0.01.

**Figure 2:**
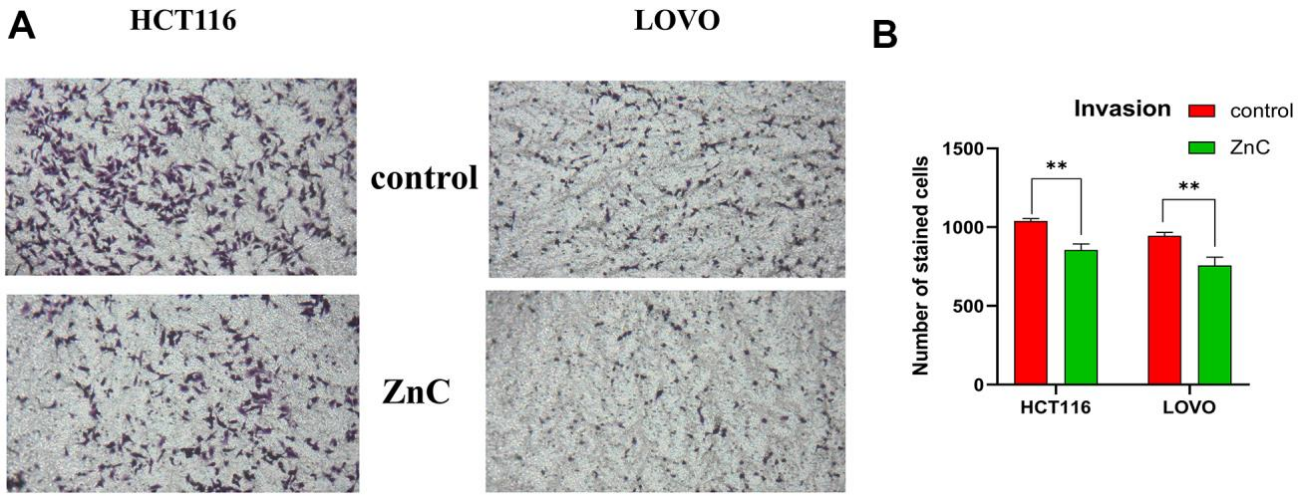
**ZnC inhibited the invasion in CRC.** (**A**) Transwell experiment was adopted to assess cell invasion of CRC cells incubated with ZnC. (**B**) Results of the cell invasion count analysis. **p < 0.01.

**Figure 3:**
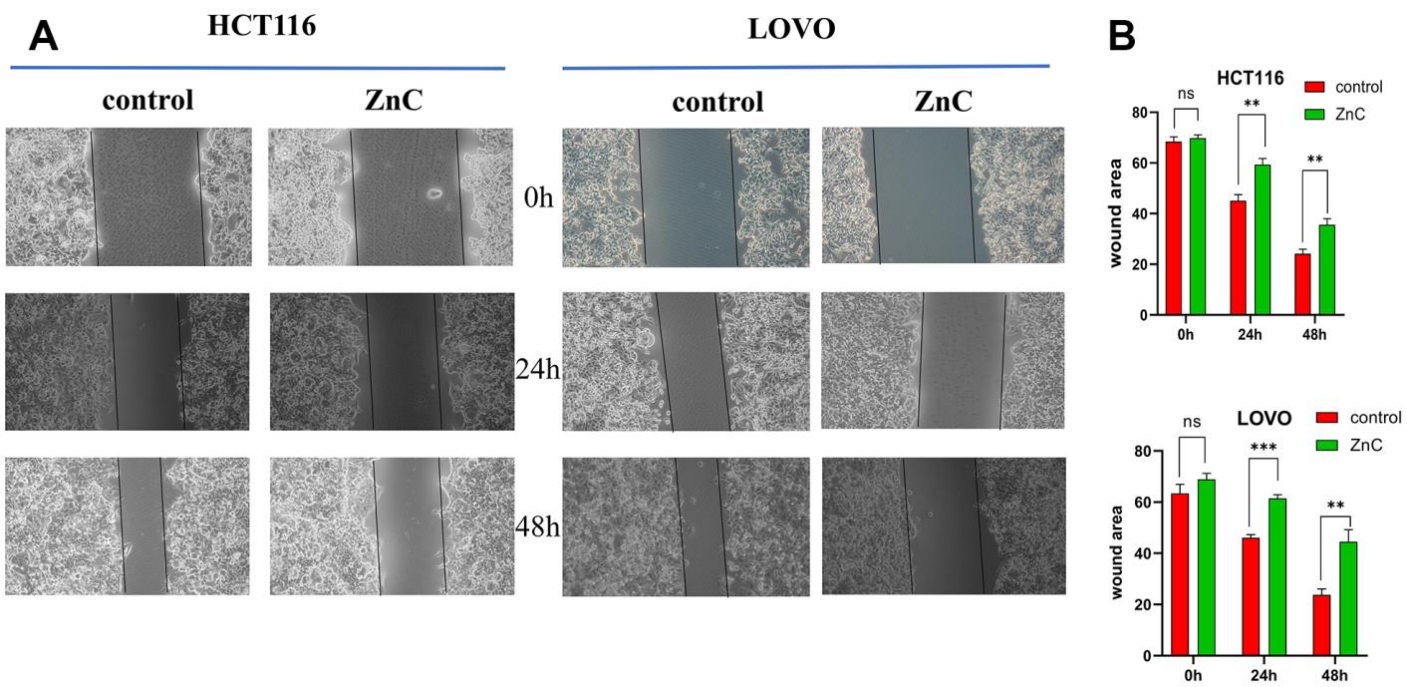
**ZnC inhibited the migration in CRC.** (**A**) Wound healing assays were used to assess cell migration of CRC cells treated by ZnC. (**B**) Results of the cell migration count analysis. **p < 0.01, ***p < 0.001.

ZnC could suppress cell proliferation in HCT116 and LOVO cells, based on the CCK-8 and EdU experiment (Figure 1A–1C). The results of transwell assay showed that ZnC inhibited the relative invasion rate of CRC cell lines compared with the control group (Figure 2A, 2B). Moreover, the wound healing assay showed that the group added with ZnC presented obviously lower scratch closure rate relative to the control group, and the closure rate decreased with the increase of incubation time (Figure 3A, 3B). To sum up, ZnC remarkably weakened the ability exhibited by HCT116 and LOVO cell lines to proliferate, invade and migrate.

### ZnC up-regulated PD-L1 expression via decreasing miR-570

**Figure 4:**
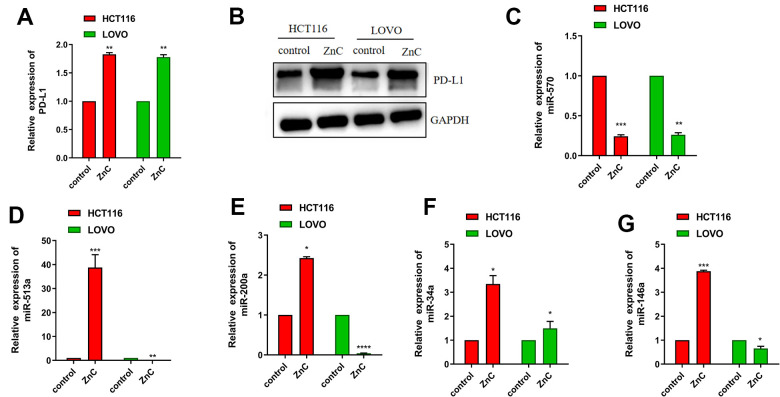
**ZnC up-regulated PD-L1 expression via decreasing miR-570.** (**A**) qRT-PCR results of PD-L1 in ZnC and PBS group. (**B**) Western blot of PD-L1 in ZnC and PBS group. (**C**–**G**) qRT-PCR results of the changes of miRNAs caused by ZnC to CRC cells. *p < 0.05, ** *P* < 0.01, ***P < 0.001.

To determine how ZnC affect the tumor immune microenvironment, we examined PD-L1 expression, which is one of the most popular target for immunotherapy currently. We surprisingly found that ZnC resulted in an increase in the PD-L1 mRNA and protein expression in CRC cells by virtue of qRT-PCR assay together with western blot (Figure 4A, 4B). The study held the purpose of confirming factors that impacted the PD-L1 increment in CRC cells due to ZnC. qRT-PCR assisted in investigating miR-570, miR-513a, miR-200a, miR-34a, and miR-146a (miRNAs) expression regarding CRC cells cultured with ZnC or PBS and results demonstrated the down regulation of miR-570 expression in HCT116 as well as LOVO cells, whereas expression of miR-513a, miR-200a, miR-34a, and miR-146a was inconsistent in the two cell lines (Figure 4C–4G). These results demonstrated that ZnC might prevent human CRC cells regarding the proliferation, invasion as well as migration, meanwhile up-regulating PD-L1 expression through down-regulating miR-570 expression.

### ZnC reduced tumor growth as well as increased the PD1 mAb treatment efficiency in xenograft mice model

**Figure 5:**
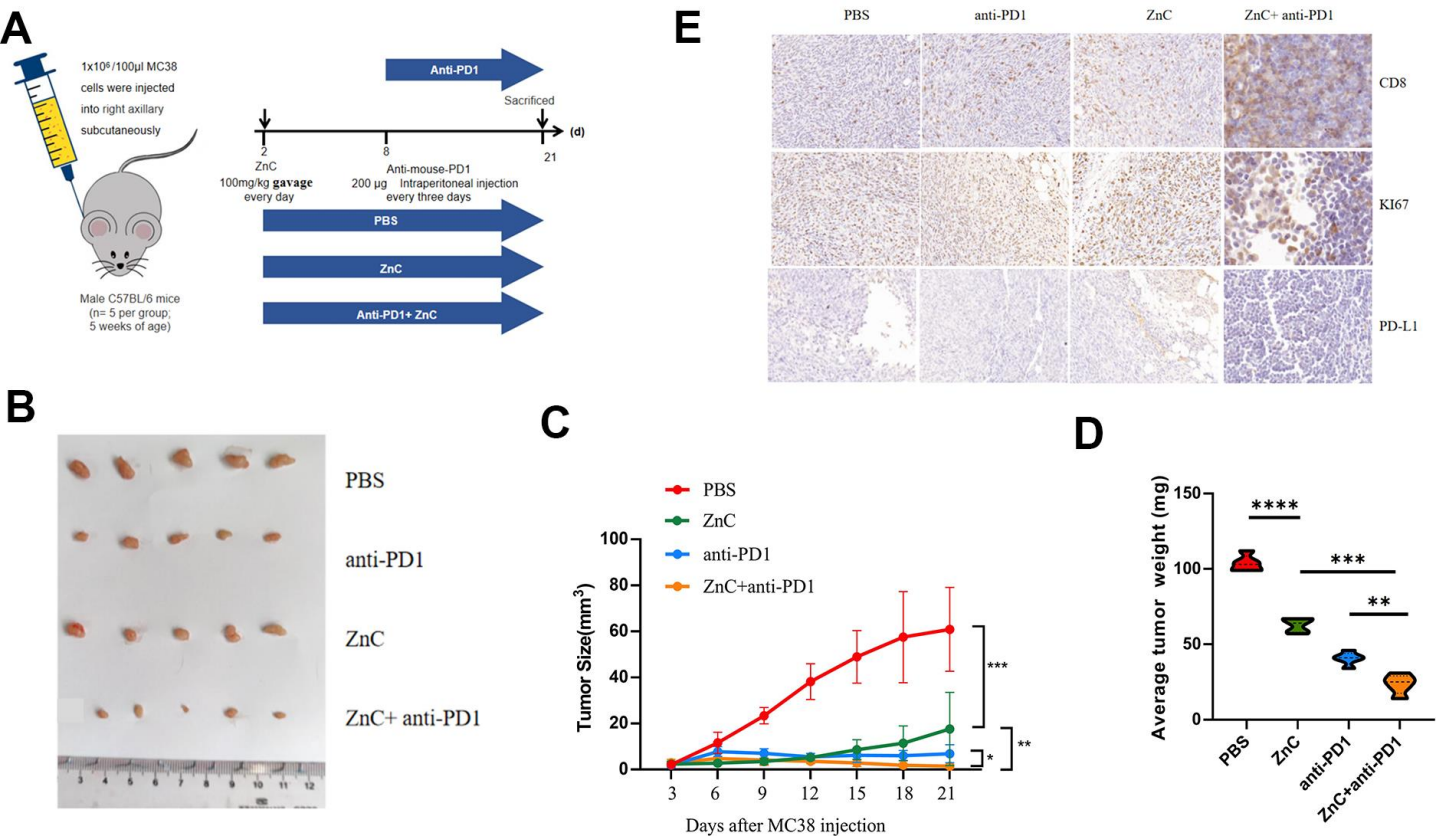
**ZnC reduced tumor growth and increased the efficiency of anti-PD1 monoclonal antibody in xenograft mice model.** (**A**) Procedures for establishing subcutaneously tumor-bearing mice model. PD1 antibody was injected internationally every 3 days. The mice injected with PBS were as the control. (**B**) Picture display of the respective group (PBS, ZnC, anti-PD1, and anti-PD1+ZnC) of subcutaneous tumors. (**C**, **D**) The volume (**C**) and weight (**D**) statistics of subcutaneous tumors in the respective group. (**E**) Immunohistochemical results of Ki67, PD-L1, and CD8 expression in the respective group. **p < 0.01, ***P < 0.001,****p < 0.0001.

*In vivo* results showed that compared with PBS group, tumor volume and weight presented an obvious decline after ZnC gavage. Compared with anti-PD1 group, combination with ZnC decreased the tumor weight and volume (Figure 5B–5D). Given the immunohistochemical results, Ki67 expression in ZnC group significantly decreased compared with PBS group, and the decline was noticeably facilitated under the combination with PD1 mAb (Figure 5E). But the CD8 and PD-L1 expression moderately increased after ZnC added. Furthermore, as revealed by immunohistochemical results, after adding ZnC, CD8 expression was extremely expanded with the combination of PD1 mAb. Hence, addition of ZnC could weaken tumor growth and enhance the PD1 mAb treatment efficiency in a xenograft mice model *in vivo*.

### Changes in tumor immune microenvironment with ZnC added *in vivo*

**Figure 6:**
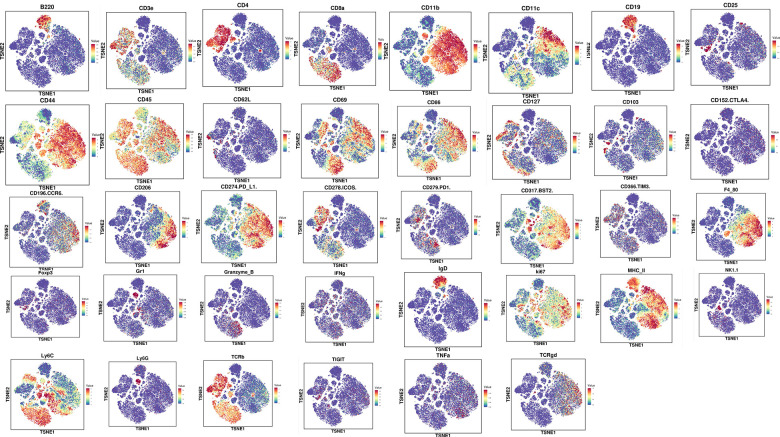
The expression of cell clustering marker genes via mass cytometry.

**Figure 7:**
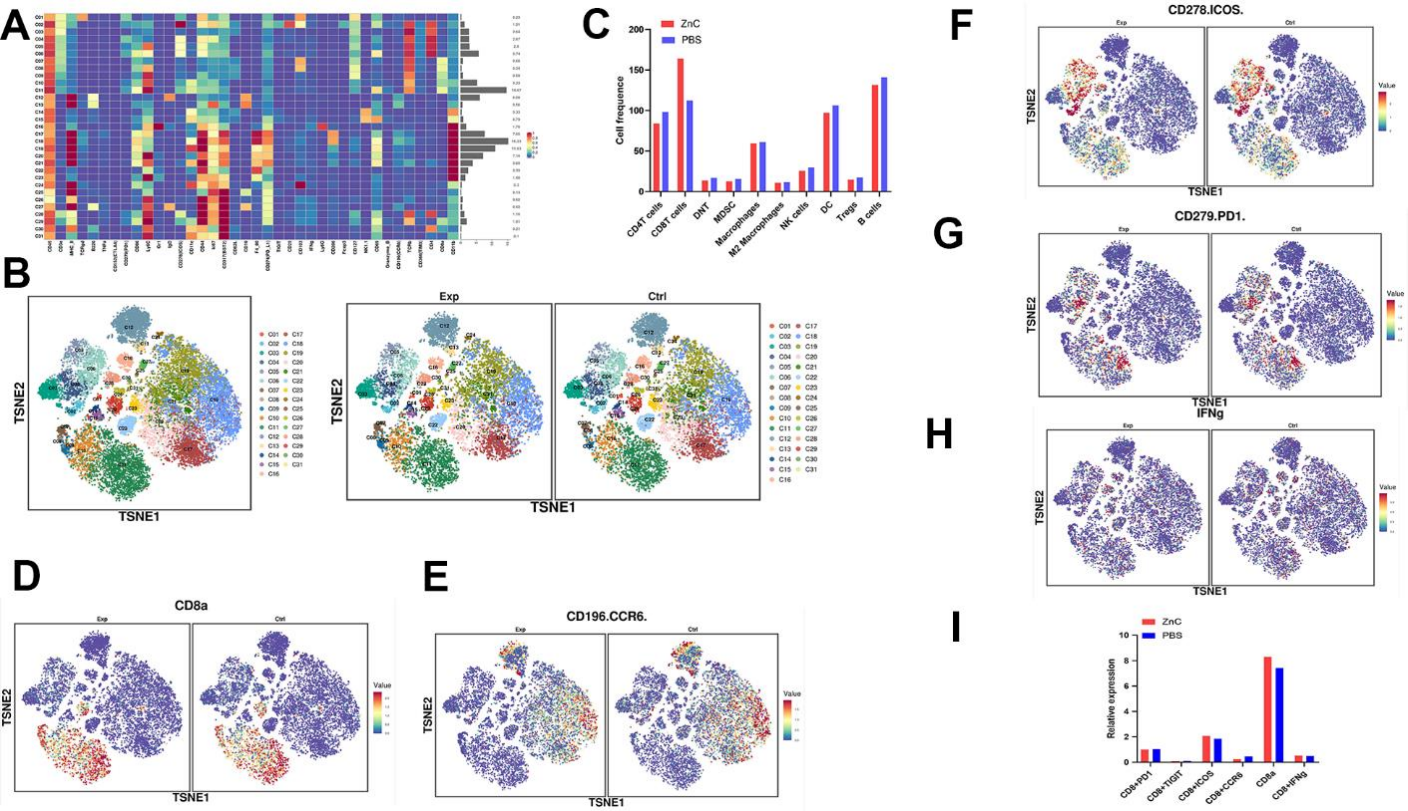
**Mass cytometry reflected the immune microenvironment of subcutaneous CRC tumors after PBS/ZnC treatment.** (**A**) A total of 31 cell clusters were divided, and we defined the respective group. (**B**) TSNE plot showing distributions of 31 cell cluster in the respective sample. (**C**) The histogram showing the number of the respective cell cluster in different groups by mass cytometry. (**D**–**H**) TSNE plot showing the distribution of CD8^+^ PD1, CD8^+^ TIGIT, CD8^+^ CCR6, CD8^+^ IFNg^,^ and CD8^+^ ICOS in subcutaneous CRC tumors in PBS and ZnC groups. (**I**) The histogram showing the expression of CD8^+^ PD1, CD8^+^ TIGIT, CD8^+^ CCR6, CD8^+^ IFNg^,^ and CD8^+^ ICOS in PBS and ZnC groups.

To further assess the overall immune microenvironment changes of CRC tumors in the PBS and ZnC groups, we measured the expression of immune cell clusters in each group using mass cytometry. There are 31 cell clusters, and we define each cluster based on specific markers for each cell type (Figures 6, 7A, 7B). The results showed that immunosuppressive cells including regulatory T cells (tregs), bone marrow-derived suppressor cells (MDSC), as well as M2 macrophages decreased whereas CD8^+^ T cells after ZnC addition (Figure 7C). Additionally, we assessed the overall expression of CD8^+^ PD1, CD8^+^ TIGIT, CD8^+^ CCR6, CD8^+^ IFNg and CD8^+^ ICOS in the immune microenvironment. Hence, after the addition of ZnC, CD8^+^ CCR6 noticeably decreased whereas CD8a and CD8^+^ ICOS expression increased (Figure 7D–7I). The mentioned results suggested that injection of ZnC could lead to the increase of CD8^+^ T cells expression, while reducing the immunosuppressive cell number in CRC.

## DISCUSSION

ZnC is usually used as a dietary zinc supplement and a potential adjunct for the treatment of peptic ulcer, taste disorders, oral mucosal repair, etc. [11]. However, ZnC is rarely used in cancer, and the biological function of ZnC and its effect on tumor immune microenvironment remain unclear. However, ZnC itself exhibits powerful anti-inflammatory and antioxidant attributes [12, 13]. According to recent studies, ZnC may assist in maintaining genomic stability as well as preventing DNA and chromosomal damage resulted from different genotoxic substances [14]. Chronic inflammation, oxidative stress, as well as genomic instability events are known to contribute to the formation of cancer [15, 16], therefore we hypothesized that ZnC has potential anticancer effects. We demonstrated that ZnC inhibited HCT116 and LOVO cell lines from the perspectives of the proliferation, invasion and migration by CCK-8, EdU, Transwell and wound healing assay. All these results indicated that ZnC could inhibit CRC cells *in vitro*, although ZnC had a moderate inhibitory effect on cancer cells. We suspect that using ZnC for a long term may make the anticancer effect even more significant. Previously, ZnC has been demonstrated to cause direct cytotoxic effects against cancer cell line as well. ZnC treating approach may inhibit the proliferation of HCC as well as assist in achieving reverse liver fibrosis [15, 16]. The *in vitro* trials are only referrals, and large clinical trials are needed to confirm whether ZnC can actually prevent cancer.

ICI, particularly anti-PD1 therapy, has greatly enlarged the therapeutic landscape of CRC recently [17–19]. However, clinical data suggest that ICIs only exert their function specific to microsatellite instability-high (MSI-H) tumors. Patients who have microsatellite stability (MSS) CRC (approx. 90%) present a response rate of only 5%-10% [20]. The above situation has limited the clinical application of PD1/PD-L1 inhibitors and caused a bottleneck in related studies. However, PD1/PD-L1 inhibitors, in combination with chemotherapy, radiotherapy, targeted drug therapy, and other immunotherapies, are capable of increasing CD8^+^ T cell number in the patient's tumor microenvironment, disrupting tumor immune escape, and enhancing the antitumor effect of PD1/PD-L1 inhibitors [18, 21]. According to REGONIVO trial, relying on the co-treatment of Regorafenib and anti-PD1 therapy, the response rate of MSS CRC patients can reach 33% [22, 23], but it is still not ideal. Therefore, improving the effect of anti-PD1 therapy is of great significance for CRC immunotherapy. Currently, many clinical trials of combined therapy are ongoing. The aim is to explore therapeutic strategies to achieve greater benefit in patients with advanced CRC with MSI-H, to break immune resistance in patients with MSS CRC, and to expand the population benefiting from this treatment approach. In a meta-analysis, PD-L1 positive patients enjoy much more clinical benefits relative to negative patients [24]. Therefore, increasing the expression regarding PD-L1 on tumor cell surface can assist in making anti-PD1 immunotherapy more sensitive.

The study confirmed that ZnC up-regulated PD-L1 expression both in mRNA and protein level, which indicated that ZnC might be an adjuvant drug for improving anti-PD1 immunotherapy in CRC. This result greatly inspired us to continue to explore its molecular mechanism. MicroRNAs (miRNAs) refer to single stranded molecules that regulate RNA, and the length is 21-23 nt. They are capable of complementing the 3’-untranslated region of target mRNA, causing the cleavage of mRNA and/or the inhibition of translation, thereby monitoring the gene expression. Based on documents, above five miRNAs are selected as the testing objects [10, 25–30]. qRT-PCR helped to demonstrate the down-regulation of miR-570 expression in HCT116 as well as LOVO cells, whereas expression of miR-513a, miR-200a, miR-34a, and miR-146a was inconsistent in the two cell lines. This does not mean that miR-513a, miR-200a, miR-34a, and miR-146a do not regulate PD-L1, but based on their consistent results in the two cell lines, miR-570 may assist in regulating PD-L1 in more CRC populations. Li-Li Wang et al. found that miR-570 targeted PD-L1 was able to inhibit breast cancer cell proliferation, invasion, migration, and induce cell apoptosis. The binding sites of miR-570 existed in the 3′-untranslated regions (3′-UTR) of PD-L1 [31]. Therefore, these results demonstrated that ZnC might prevent human CRC cells from the perspectives of proliferation, invasion and migration, and up-regulate PD-L1 expression through down-regulating miR-570 expression.

Based on the *in vivo* experiments, we confirmed that CD8^+^ T cell expression presented a moderate increase after adding ZnC via both immunohistochemistry and mass cytometry. The results also showed that immunosuppressive cells including tregs, MDSC, and M2 macrophages decreased after the addition of ZnC. M2 macrophages can generate angiogenic factors, like VEGF, PDGF, and matrix metalloproteinases (MMPs), meanwhile inducing neovascularization [32, 33] Various secretory factors can mediate the immunosuppressive function possessed by MDSCs, like prolandin E2 (PGE2), IL-10, TGF-β, nitric oxide (NO) and arginase 1 (Arg1) [34], as well as ROS, G-CSF and Arg1 for granulocytic-MDSCs [35]. MDSCs are capable of secreting MMP9 for promoting the anti-PD1 resistance cleavage of PD-L1 surface expression, which is dependent of MMP9 [36], and hypoxia restricts the anti-tumor function exhibited by anti-PD1 Abs. Tregs express multiple immune checkpoints, inhibit the cytotoxic function as well as proliferation regarding conventional effector T cells, meanwhile assisting in maintaining an immunosuppressive tumor microenvironment. The above analysis shows that reducing immunosuppressive cells is beneficial to create a tumor microenvironment that is conducive to anti-PD1 immunotherapy.

Our results also demonstrate that ZnC is capable of promoting CD8^+^ T cell infiltration in tumors. All of these findings enrich the function of ZnC to assist in enhancing anti-PD1 immune efficacy in CRC. Additionally, we assessed the overall expression of CD8^+^ PD1, CD8^+^ TIGIT, CD8^+^ CCR6, CD8^+^ IFNg and CD8^+^ ICOS in the immune microenvironment. As found, after the addition of ZnC, CD8^+^ CCR6 noticeably decreased whereas CD8^+^ ICOS expression increased. ICOS presents up-regulation in the activated T lymphocytes, primarily after the anti-CTLA-4 therapies [37]. As revealed by Fan et al., by blocking CTLA-4 and participating in ICOS with tumor cell vaccine, engineered expression of ICOS ligand resulted in a qualitative and quantitative increase in the anti-tumor immune response in mice that had developed prostate cancer and melanoma significantly promoted rejection [38]. A study that assessed the CCR6 expression in colon cancer detected CCR6 expression in samples from colon cancer patients for the first time and revealed its higher expression in colon cancer relative to normal controls. Furthermore, CCR6 expression presented a positive relation to lymph node status and metastasis. Actually, CCR6 is most highly expressed in metastatic colon cancer cells [39]. These results fully demonstrated that anti-PD1 therapy was more sensitive in a tumor microenvironment with CD8^+^ T cell enrichment, decreased immunosuppressive cells, and elevated PD-L1 on the tumor cell surface. However, there are too few samples detected by mass cytometry and the current results can only represent a trend, and more samples need to be verified in the future. We look forward to more research on the combination of drugs and PD1 mAbs flourishing in the field of cancer immunotherapy.

## CONCLUSIONS

**Figure 8:**
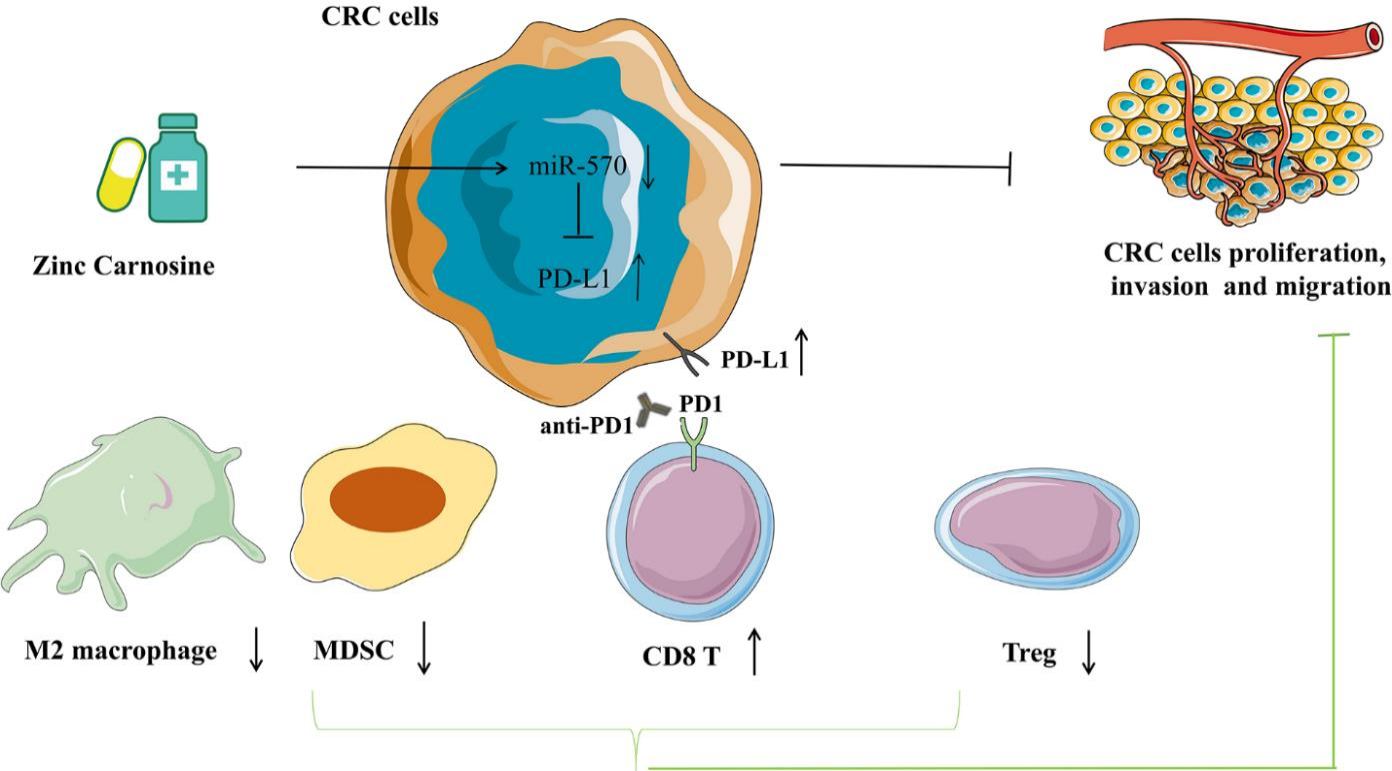
**Pattern diagram showing that ZnC slows the progression of CRC by inhibiting the proliferation, invasion and migration and up-regulates PD-L1 expression via inhibiting miR-570.** The combination of ZnC and anti-PD1 therapy is beneficial to synergically increase efficacy of anti-tumor in CRC therapy.

The present study reveals that ZnC slows the progression of CRC by inhibiting the proliferation, invasion and migration and up-regulates PD-L1 expression via inhibiting miR-570. The ZnC-anti-PD1 co-treatment assists in synergically strengthening the efficacy exhibited by anti-tumor in CRC therapy (Figure 8).

## MATERIALS AND METHODS

### Cell cultures and drug treatment

The Type culture bank of Chinese Academy of Sciences provided human CRC cell lines (HCT116 and LOVO) as well as mouse CRC cell lines (MC38). HCT116 and LOVO cells underwent culturing treatment in DMEM (Adding 10% FBS); 50 U /mL penicillin and 50mg /mL streptomycin (Gibco, USA). MC38 cells were cultured in RPMI 1640 medium (Gibco, USA) that contained 10% FBS, 50 U/mL penicillin and 50 mg/mL streptomycin. We cultured all cell lines in an incubator at 37° C and 5% CO_2_.

### Cell proliferation experiments

We placed HCT116 and LOVO cells in 96-well plates (2000 cells/well), treating them using 10μ L CCK8 solution (RiboBio, China). ZnC (Polaprezinc, Selleck, USA,10 mM) was applied to cells at 0h, 24h, 48h, 72h and 96h respectively. Subsequently, we studied the absorbance of cells at 450 nm when employing a microplate reading element as per the instruction of manufacturer (Synergy, USA). Using the Cell-light EdU DNA Cell Proliferation Kit (RiboBio, China), we performed EdU experiments for evaluating Cell proliferation. First, 8 × 10^4^ CRC cells were electroplated on 24-well plates, and ZnC (10 mM) treatment was performed for 24h when the cells reached 80-90% fusion degree. Then both cell lines were fixed using 4% paraformaldehyde, followed by one day of incubation in 10mmol/L EdU solution. Finally, cell lines received the treatment of Apollo Dye Solution and DAPI respectively as per the manufacturer's procedures. Olympus FSX100 microscope (Olympus, Japan) assisted in counting the collected EdU merged cells.

### Transwell invasion assay

HCT116 and LOVO cells (3 ×10^5^ cells) were respectively tiled into the upper chamber containing 300μl serum-free medium, with ZnC (10 mM) pretreating for 24h. The transwell chamber was laid with matrix mixtures (BD Biosciences, USA) for the invasion tests. We used 600μl complete medium with 10% FBS to fill the bottom cavity, which is a chemical attractant specific to CRC cells. Following 24-hour incubation, the cells invading the outer membrane underwent 10 minutes of fixing treatment in 4% paraformaldehyde, followed by 15 min of staining using Crystal Violet Staining Solution (Beyotime, China). Subsequently, the stained cells were photographed under the microscope and counted for analysis.

### Wound healing assay

We placed HCT116 and LOVO cells on 6-well culture plates and these cells grew into cell monolayers with 90 - 100% confluence. Then, the monolayers were scratched by a standard 10μl pipette tip. Phosphate Buffered Saline (PBS, Gibco, USA) served for slightly removing the free floating cells as well as debris. Afterwards, serum-free medium containing 10 mM concentration of ZnC was added, and the plate received incubation at 37 normal temperature. The inverted microscope recorded the widths of the scratch gap after 0h, 24h and 48h.

### Quantitative reverse transcription polymerase reaction (qRT-PCR)

TRIzol reagent served for isolating total RNA from cells according to Invitrogen's manufacturing process. CDNA and miRNAs of PD-L1 were synthesized using reverse transcription kit (Takara, Japan) together with RiboBio (RiboBio, China). Human PD-L1 primer sequences were 5' -TCACTTGGTAATTCTGGGAGC-3 '(forward) and 5'-CTTTGAGTTTGTATCTTGGATGCC-3 '(backward). GAPDH and U6 were adopted for normalizing the expressions of PD-L1 and miRNA before calculation.

### *In vivo* tumor mice model and treatment

Animal experiments are completed with the approval of the Animal Management Committee of Nanjing Medical University, and all experimental procedures and animal care conform to the institutional ethics of animal experiments. Twenty male C57BL/6 mice (5 weeks old) came from the Experimental Animal Center of Nanjing Medical University and reared under the specific pathogen free (SPF) conditions. For building subcutaneous tumor-bearing mouse model, C57BL/6 mice received subcutaneous inoculation with 1×10^6^ murine MC38 cells mixed in 100μl PBS in the right axillary region. After that, every 5 mice were randomly assigned to one group. Different groups were as follows: PBS, ZnC, anti-PD1, and anti-PD1+ZnC. On the second day, ZnC (100 mg/kg, once every day) was gavaged into the mice in the ZnC and anti-PD1+ZnC groups. After 8 days, the mice in anti-PD1 and anti-PD1+ZnC groups underwent intraperitoneal injection with 200ug anti-mouse-PD1 (BE0273; Bio X Cell, USA) every three days. We measured the tumor size each 3 days and calculated the tumor volume based on the formula: volume (mm^3^) = width^2^ × length/2. On day 21, we killed the mice using cervical dislocation, and examined the tumor samples for further analysis (Figure 5A).

### Immunohistochemistry

Subcutaneous tumor specimens of CRC mouse models were taken, paraffin embedded, sliced to 4mm thickness, immunohistochemistry was performed. 0.01 mol/L citric acid buffer solution (pH 6.0; Kaigen, China). At room temperature, we enclosed the samples in PBS containing 2% BSA for one hour and then treated them using CD8 mAb (Abcam, UK), Ki-67 (Abcam, UK) and PD-L1 (Abcam, UK) 4° C, then enzyme-conjugate secondary antibody (Abcam, UK) at room temperature for 1 hour. The next day 3'-diaminobenzidine (DAB; Kaigen, China) was administered, according to manufacturer's instructions. A laser scanning confocal microscope (Zeiss, Germany) served for capturing images.

### Mass cytometry

The tissue samples were obtained from the PBS and ZnC tumor-bearing groups. We treated mouse tumor tissue with the Miltenyi Mouse Tumor Isolation Kit (Miltenyi Biotec, Germany), and Percoll removed debris and divided red blood cells. We carried out the experiment in PLTTECH (China).

### Statistical analysis

GraphPad Prism 8.0 software served for statistical analysis. All data are in the form of mean ± SD unless otherwise stated. Unless otherwise noted, the 2 tail-student *t* test was used to compare 2 independent samples. ANOVA, 2-tailed, is used to determine variation between or between groups unless otherwise noted. A P < 0.05 reported statistical significance.

## References

[1] Rawla P, Sunkara T, Barsouk A. Epidemiology of colorectal cancer: incidence, mortality, survival, and risk factors. Prz Gastroenterol. 2019; 14:89–103. 10.5114/pg.2018.81072 31616522 PMC6791134

[2] Hirano H, Takashima A, Hamaguchi T, Shida D, Kanemitsu Y, and Colorectal Cancer Study Group (CCSG) of the Japan Clinical Oncology Group (JCOG). Current status and perspectives of immune checkpoint inhibitors for colorectal cancer. Jpn J Clin Oncol. 2021; 51:10–9. 10.1093/jjco/hyaa200 33205813

[3] Sharma P, Hu-Lieskovan S, Wargo JA, Ribas A. Primary, Adaptive, and Acquired Resistance to Cancer Immunotherapy. Cell. 2017; 168:707–23. 10.1016/j.cell.2017.01.017 28187290 PMC5391692

[4] Zappasodi R, Merghoub T, Wolchok JD. Emerging Concepts for Immune Checkpoint Blockade-Based Combination Therapies. Cancer Cell. 2018; 33:581–98. 10.1016/j.ccell.2018.03.005 29634946 PMC5896787

[5] Wargo JA, Reuben A, Cooper ZA, Oh KS, Sullivan RJ. Immune Effects of Chemotherapy, Radiation, and Targeted Therapy and Opportunities for Combination With Immunotherapy. Semin Oncol. 2015; 42:601–16. 10.1053/j.seminoncol.2015.05.007 26320064 PMC4955940

[6] Ingles Garces AH, Au L, Mason R, Thomas J, Larkin J. Building on the anti-PD1/PD-L1 backbone: combination immunotherapy for cancer. Expert Opin Investig Drugs. 2019; 28:695–708. 10.1080/13543784.2019.1649657 31359805

[7] Li Z, Sun G, Sun G, Cheng Y, Wu L, Wang Q, Lv C, Zhou Y, Xia Y, Tang W. Various Uses of PD1/PD-L1 Inhibitor in Oncology: Opportunities and Challenges. Front Oncol. 2021; 11:771335. 10.3389/fonc.2021.771335 34869005 PMC8635629

[8] Choi HS, Kim ES, Keum B, Chun HJ, Sung MK. L-carnosine and zinc in gastric protection. Imidazole Dipeptides. 2015; 548–65. 10.1039/9781782622611-00548

[9] Shimada T, Watanabe N, Ohtsuka Y, Endoh M, Kojima K, Hiraishi H, Terano A. Polaprezinc down-regulates proinflammatory cytokine-induced nuclear factor-kappaB activiation and interleukin-8 expression in gastric epithelial cells. J Pharmacol Exp Ther. 1999; 291:345–52. 10490923

[10] Hewlings S, Kalman D. A Review of Zinc-L-Carnosine and Its Positive Effects on Oral Mucositis, Taste Disorders, and Gastrointestinal Disorders. Nutrients. 2020; 12:665. 10.3390/nu12030665 32121367 PMC7146259

[11] Ackland ML, Michalczyk AA. Zinc and infant nutrition. Arch Biochem Biophys. 2016; 611:51–7. 10.1016/j.abb.2016.06.011 27317042

[12] Prasad AS. Zinc in human health: effect of zinc on immune cells. Mol Med. 2008. 10.2119/2008-00033.Prasad 18385818 PMC2277319

[13] Prasad AS. Impact of the discovery of human zinc deficiency on health. J Am Coll Nutr. 2009; 28:257–65. 10.1080/07315724.2009.10719780 20150599

[14] Ooi TC, Chan KM, Sharif R. Protective effects of zinc L-carnosine against hydrogen peroxide-induced DNA damage and micronucleus formation in CCD-18co human colon fibroblast cells. Free Radic Res. 2020; 54:330–40. 10.1080/10715762.2020.1763333 32366187

[15] Ooi TC, Chan KM, Sharif R. Zinc L-Carnosine Protects CCD-18co Cells from L-Buthionine Sulfoximine-Induced Oxidative Stress via the Induction of Metallothionein and Superoxide Dismutase 1 Expression. Biol Trace Elem Res. 2020; 198:464–71. 10.1007/s12011-020-02108-9 32146577

[16] Hayashi H, Kobayashi R, Suzuki A, Yamada Y, Ishida M, Shakui T, Kitagawa J, Hayashi H, Sugiyama T, Takeuchi H, Tsurumi H, Itoh Y. Preparation and clinical evaluation of a novel lozenge containing polaprezinc, a zinc-L-carnosine, for prevention of oral mucositis in patients with hematological cancer who received high-dose chemotherapy. Med Oncol. 2016; 33:91. 10.1007/s12032-016-0795-z 27418192 PMC4945687

[17] Payandeh Z, Khalili S, Somi MH, Mard-Soltani M, Baghbanzadeh A, Hajiasgharzadeh K, Samadi N, Baradaran B. PD-1/PD-L1-dependent immune response in colorectal cancer. J Cell Physiol. 2020; 235:5461–75. 10.1002/jcp.29494 31960962

[18] Patel SA, Minn AJ. Combination Cancer Therapy with Immune Checkpoint Blockade: Mechanisms and Strategies. Immunity. 2018; 48:417–33. 10.1016/j.immuni.2018.03.007 29562193 PMC6948191

[19] Overman MJ, McDermott R, Leach JL, Lonardi S, Lenz HJ, Morse MA, Desai J, Hill A, Axelson M, Moss RA, Goldberg MV, Cao ZA, Ledeine JM, et al. Nivolumab in patients with metastatic DNA mismatch repair-deficient or microsatellite instability-high colorectal cancer (CheckMate 142): an open-label, multicentre, phase 2 study. Lancet Oncol. 2017; 18:1182–91. 10.1016/S1470-2045(17)30422-9 28734759 PMC6207072

[20] Le DT, Durham JN, Smith KN, Wang H, Bartlett BR, Aulakh LK, Lu S, Kemberling H, Wilt C, Luber BS, Wong F, Azad NS, Rucki AA, et al. Mismatch repair deficiency predicts response of solid tumors to PD-1 blockade. Science. 2017; 357:409–13. 10.1126/science.aan6733 28596308 PMC5576142

[21] Hsu JM, Li CW, Lai YJ, Hung MC. Posttranslational Modifications of PD-L1 and Their Applications in Cancer Therapy. Cancer Res. 2018; 78:6349–53. 10.1158/0008-5472.CAN-18-1892 30442814 PMC6242346

[22] Chen J, Ye X, Pitmon E, Lu M, Wan J, Jellison ER, Adler AJ, Vella AT, Wang K. IL-17 inhibits CXCL9/10-mediated recruitment of CD8^+^ cytotoxic T cells and regulatory T cells to colorectal tumors. J Immunother Cancer. 2019; 7:324. 10.1186/s40425-019-0757-z 31775909 PMC6880503

[23] Fukuoka S, Hara H, Takahashi N, Kojima T, Kawazoe A, Asayama M, Yoshii T, Kotani D, Tamura H, Mikamoto Y, Hirano N, Wakabayashi M, Nomura S, et al. Regorafenib Plus Nivolumab in Patients With Advanced Gastric or Colorectal Cancer: An Open-Label, Dose-Escalation, and Dose-Expansion Phase Ib Trial (REGONIVO, EPOC1603). J Clin Oncol. 2020; 38:2053–61. 10.1200/JCO.19.03296 32343640

[24] Shen X, Zhao B. Efficacy of PD-1 or PD-L1 inhibitors and PD-L1 expression status in cancer: meta-analysis. BMJ. 2018; 362:k3529. 10.1136/bmj.k3529 30201790 PMC6129950

[25] Shadbad MA, Safaei S, Brunetti O, Derakhshani A, Lotfinejad P, Mokhtarzadeh A, Hemmat N, Racanelli V, Solimando AG, Argentiero A, Silvestris N, Baradaran B. A Systematic Review on the Therapeutic Potentiality of PD-L1-Inhibiting MicroRNAs for Triple-Negative Breast Cancer: Toward Single-Cell Sequencing-Guided Biomimetic Delivery. Genes (Basel). 2021; 12:1206. 10.3390/genes12081206 34440380 PMC8391239

[26] Wei S, Wang K, Huang X, Zhao Z, Zhao Z. LncRNA MALAT1 contributes to non-small cell lung cancer progression via modulating miR-200a-3p/programmed death-ligand 1 axis. Int J Immunopathol Pharmacol. 2019. 10.1177/2058738419859699 31240979 PMC6595645

[27] Li J, Che L, Xu C, Lu D, Xu Y, Liu M, Chai W. XIST/miR-34a-5p/PDL1 axis regulated the development of lung cancer cells and the immune function of CD8^+^ T cells. J Recept Signal Transduct Res. 2022; 42:469–78. 10.1080/10799893.2021.2019274 35067156

[28] Søndergaard HB, Airas L, Christensen JR, Nielsen BR, Börnsen L, Oturai A, Sellebjerg F. Pregnancy-Induced Changes in microRNA Expression in Multiple Sclerosis. Front Immunol. 2021; 11:552101. 10.3389/fimmu.2020.552101 33584638 PMC7876450

[29] Di Raimondo C, Han Z, Su C, Wu X, Qin H, Sanchez JF, Yuan YC, Martinez X, Abdulla F, Zain J, Chen CW, Rosen ST, Querfeld C. Identification of a Distinct miRNA Regulatory Network in the Tumor Microenvironment of Transformed Mycosis Fungoides. Cancers (Basel). 2021; 13:5854. 10.3390/cancers13225854 34831008 PMC8616450

[30] Chen J, Jiang CC, Jin L, Zhang XD. Regulation of PD-L1: a novel role of pro-survival signalling in cancer. Ann Oncol. 2016; 27:409–16. 10.1093/annonc/mdv615 26681673

[31] Wang LL, Huang WW, Huang J, Huang RF, Li NN, Hong Y, Chen ML, Wu F, Liu J. Protective effect of hsa-miR-570-3p targeting CD274 on triple negative breast cancer by blocking PI3K/AKT/mTOR signaling pathway. Kaohsiung J Med Sci. 2020; 36:581–91. 10.1002/kjm2.12212 32311203 PMC11896444

[32] Repasky EA, Evans SS, Dewhirst MW. Temperature matters! And why it should matter to tumor immunologists. Cancer Immunol Res. 2013; 1:210–6. 10.1158/2326-6066.CIR-13-0118 24490177 PMC3904378

[33] Wang T, Ge Y, Xiao M, Lopez-Coral A, Azuma R, Somasundaram R, Zhang G, Wei Z, Xu X, Rauscher FJ 3rd, Herlyn M, Kaufman RE. Melanoma-derived conditioned media efficiently induce the differentiation of monocytes to macrophages that display a highly invasive gene signature. Pigment Cell Melanoma Res. 2012; 25:493–505. 10.1111/j.1755-148X.2012.01005.x 22498258 PMC3615702

[34] Bergenfelz C, Leandersson K. The Generation and Identity of Human Myeloid-Derived Suppressor Cells. Front Oncol. 2020; 10:109. 10.3389/fonc.2020.00109 32117758 PMC7025543

[35] Najafi M, Goradel NH, Farhood B, Salehi E, Solhjoo S, Toolee H, Kharazinejad E, Mortezaee K. Tumor microenvironment: Interactions and therapy. J Cell Physiol. 2019; 234:5700–21. 10.1002/jcp.27425 30378106

[36] Ohue Y, Nishikawa H. Regulatory T (Treg) cells in cancer: Can Treg cells be a new therapeutic target? Cancer Sci. 2019; 110:2080–9. 10.1111/cas.14069 31102428 PMC6609813

[37] Sanmamed MF, Pastor F, Rodriguez A, Perez-Gracia JL, Rodriguez-Ruiz ME, Jure-Kunkel M, Melero I. Agonists of Co-stimulation in Cancer Immunotherapy Directed Against CD137, OX40, GITR, CD27, CD28, and ICOS. Semin Oncol. 2015; 42:640–55. 10.1053/j.seminoncol.2015.05.014 26320067

[38] Fan X, Quezada SA, Sepulveda MA, Sharma P, Allison JP. Engagement of the ICOS pathway markedly enhances efficacy of CTLA-4 blockade in cancer immunotherapy. J Exp Med. 2014; 211:715–25. 10.1084/jem.20130590 24687957 PMC3978270

[39] Kapur N, Mir H, Clark Iii CE, Krishnamurti U, Beech DJ, Lillard JW, Singh S. CCR6 expression in colon cancer is associated with advanced disease and supports epithelial-to-mesenchymal transition. Br J Cancer. 2016; 114:1343–51. 10.1038/bjc.2016.113 27149649 PMC4984452

